# NOA: a cytoscape plugin for network ontology analysis

**DOI:** 10.1093/bioinformatics/btt334

**Published:** 2013-06-07

**Authors:** Chao Zhang, Jiguang Wang, Kristina Hanspers, Dong Xu, Luonan Chen, Alexander R. Pico

**Affiliations:** ^1^Department of Computer Science and Christopher S. Bond Life Sciences Center, University of Missouri, Columbia, MO 65211, USA, ^2^Beijing Institute of Genomics, 100029 and ^3^Academy of Mathematics and Systems Science, Chinese Academy of Sciences, Beijing 100190, China, ^4^Gladstone Institutes, San Francisco, CA 94158, USA and ^5^Shanghai Institute for Biological Science, Chinese Academy of Sciences, 200031 China

## Abstract

**Summary:** The Network Ontology Analysis (NOA) plugin for Cytoscape implements the NOA algorithm for network-based enrichment analysis, which extends Gene Ontology annotations to network links, or edges. The plugin facilitates the annotation and analysis of one or more networks in Cytoscape according to user-defined parameters. In addition to tables, the NOA plugin also presents results in the form of heatmaps and overview networks in Cytoscape, which can be exported for publication figures.

**Availability:** The NOA plugin is an open source, Java program for Cytoscape version 2.8 available via the Cytoscape App Store (http://apps.cytoscape.org/apps/noa) and plugin manager. A detailed user manual is available at http://nrnb.org/tools/noa.

**Contact:** apico@gladstone.ucsf.edu

**Supplementary information:** Supplementary data are available at *Bioinformatics* online.

## 1 INTRODUCTION

To make use of the vast wealth of data and knowledge to elucidate the function of biological networks is one of the biggest challenges in bioinformatics. The development of high-throughput technology has given rise to an enormous increase of data on biomolecular expression and interactions, which results in protein interaction networks, gene regulatory networks, signaling networks and metabolic networks. One approach to understanding networks relies on ontologies, such as Gene Ontology (GO), and enrichment analysis. Common approaches, such as BiNGO (Maere *et al.*, 2005), regard a network as a list of genes, performing gene-level annotation and enrichment methods. However, it is apparent that the same list of genes with different interactions may perform different functions. Thus, the functional analysis of networks should take into consideration network topology.

Our previous work introduced the Network Ontology Analysis (NOA) algorithm to perform gene ontology enrichment analysis on biological networks (Wang *et al.*, 2011). First, NOA assigns ontology terms to interactions based on the known annotations of connected genes via optimizing two novel indexes ‘Coverage’ and ‘Diversity’. Then, NOA generates two alternative reference sets to statistically rank the enriched functional terms for a given biological network. NOA was shown to be more efficient not only for dynamic regulatory networks but also for rewired protein interaction networks. However, there are several shortcomings with the initial implementation: no graphical interface or visualization of results; limited support for species, gene names and ontology types; no batch mode for large-scale computation; and no integration or interoperability with other network tools. To overcome these shortcomings, we have reimplemented the NOA algorithm as a Cytoscape plugin and added interfaces, extensible ontology and identifier support, new visualizations, a batch mode and interoperability with other Cytoscape plugins, as demonstrated with CyThesaurus (van Iersel *et al.*, 2010) and Mosaic (Zhang *et al.*, 2012).

## 2 METHODS

### 2.1 Algorithms

The original NOA algorithm was described in (Wang *et al.*, 2011). In the NOA plugin, we have enhanced the network annotation algorithm to include standard statistical enrichment methods (e.g. hypergeometric distribution, Fisher’s exact test and Z-score). We also added the option of running a conventional node-based analysis (i.e. gene set enrichment), to complement the edge-based approach. Several widely used correction methods have been used to control the type I error rate of multiple hypotheses testing, such as Bonferroni and Benjiamini & Hochberg methods.

Taking advantage of data model and interoperability within Cytoscape, users can perform NOA analysis on any network opened in Cytoscape or any subnetwork, selected manually, by built-in filtering mechanisms, or even by another plugin. The NOA plugin also has multiple options for reference networks based on the selected algorithm and test network.

### 2.2 Batch mode

Batch mode is another unique feature of the NOA plugin, allowing for the simultaneous analysis of multiple networks. Batch mode recognizes network and gene set file formats and selects an appropriate algorithm (edge-based or node-based) accordingly. In the case of edge-based analysis, each network is analyzed with respect to the combination of all input networks or their derived clique (connecting all nodes). Node-based analysis uses either the combination of all input genes or a given genome as reference.

In batch mode, the primary output is a ranked list of GO terms significantly overrepresented for each network based on the selected algorithm. The result list is displayed as an annotated table, including GO term type, *P* value, description and associated nodes/edges. An additional table displays the list of GO terms enriched in multiple networks in the batch. The plugin also generates a sorted heatmap showing the most significantly enriched GO terms against all input networks. These tables and heatmap can be exported to third-party tools for custom visualization and analysis.

### 2.3 Single mode

In single mode, the NOA plugin can analyze any network in Cytoscape. For edge-based analysis, the clique form of the selected network is used as reference by default. If the user selects a subnetwork for analysis, then they also have the option of using the original parent network as reference. In the case of node-based analysis, the corresponding genome is used as reference, or the complete gene set from a given network if only a subset is selected for analysis.

The results include a ranked and annotated list of GO terms, similar to batch mode. And, unique to single mode, an overview network is generated with significant GO terms represented as nodes. The size of these nodes is based on the number of occurrences of a given GO term annotation on the edges (or nodes) of the analyzed network. The co-occurrence of any two GO terms is represented by edges in the overview network.

## 3 DISCUSSION

The following use cases demonstrate the analysis options available in the NOA plugin and the types of the results users should expect.

### 3.1 Human pathways

**Fig. 1.**
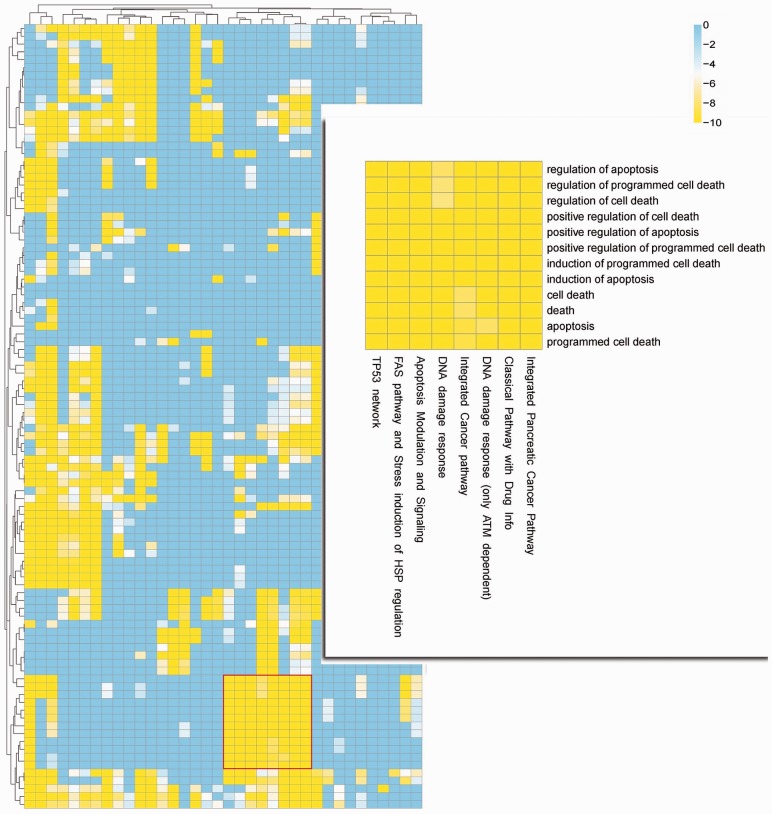
A heatmap of GO-annotated human pathways based on batch mode NOA plugin analysis. The gradient presents the corresponding logarithmic *P* value from −10 to 0. Axes are labeled in S2 and inset, highlighting apoptosis-related pathways and GO terms

We prepared a file containing all the human pathways curated at WikiPathways (Kelder *et al.*, 2012) and translated Ensembl identifiers for batch mode analysis by the NOA plugin (Supplementary File S1). We ran edge-based analysis to make use of the interaction content in the pathways, rather than just treating them like disconnected gene sets. Each pathway was analyzed against the entire collection as reference and no correction was applied. As expected, curated pathways of known function were significantly annotated with relevant GO terms (Fig. 1 and Supplementary Fig. S2). Users could add novel pathways or interaction networks to this batch analysis to assess functional annotation of edges in the context of known biology.

### 3.2 Human diseases

We collected protein interaction networks for different types of genetic diseases constructed using a generic protein–protein interactome as the source of disease gene interactions (Wang *et al.*, 2009) (Supplementary File S3). We compared edge-based and node-based analyses using the NOA plugin in batch mode. The ranked and annotated lists of enriched GO terms in Supplementary Tables S4 and S5 show many disease-term pairings in common, not surprisingly, but also a few differences. Significantly, the inclusion of generic (not disease-specific) interactions did not lead to less specific or aberrant GO terms with the edge-based method. Thus, with the NOA plugin, users can easily run both edge- and node-based analyses on gene sets of interest, using interactome data to supply interactions.

### 3.3 Yeast cell cycle

We collected time-series microarray data on yeast cell cycle and constructed regulatory transcription networks for each time point using transcription factor-target pairs revealed by ChIP-chip experiments (Supplementary File S6). Once again, we compared both types of analysis and two types of reference networks in batch analysis using the NOA plugin (Supplementary Fig. S7).

Edge-based analysis provided more specific GO terms than node-based and reflected the known biological processes. We also noted that the all-network reference produced better results (i.e. more dynamic range and in line with known biology) than the clique-based reference. When working with densely connected networks, like in this example, users will typically produce better results using the all-network option as reference.

*Funding*: National Institutes of Health [NRNB P41-GM103504 to A.R.P., R01-GM100701 to D.X.], NSFC [91029301 to L.C.], the Google Summer of Code program [C.Z.] and NSFC [11131009 to J.W.].

*Conflict of interest:* None declared.

## Supplementary Material

Supplementary Data
