# Supplementary material for: NOA: a cytoscape plugin for network ontology analysis
Source: Bioinformatics. 2013 Jun 7;29(16):2066–7. doi: 10.1093/bioinformatics/btt334 (PMC3722524; doi:10.1093/bioinformatics/btt334)
Supplement: Supplementary Data [file supp_29_16_2066__index.html]

NOA: a cytoscape plugin for network ontology analysis — NOA: a cytoscape plugin for network ontology analysis — Supplementary Data 

# NOA: a cytoscape plugin for network ontology analysis

## 

files

**Files in this Data Supplement:**

- Supplementary Data - pdf file
- Supplementary Data - pdf file
- Supplementary Data - txt file
- Supplementary Data - png file
- Supplementary Data - txt file
- Supplementary Data - txt file
- Supplementary Data - png file
