# Supplementary material for: NOA: a cytoscape plugin for network ontology analysis
Source: Bioinformatics. 2013 Jun 7;29(16):2066–7. doi: 10.1093/bioinformatics/btt334 (PMC3722524; doi:10.1093/bioinformatics/btt334)
Supplement: Supplementary Data [file supp_btt334_S4.pdf]

Supplementary Table S4

Edge-based, All network, Corrected				
Disease	GO ID	Type	P-value	Description
Cancer	GO:0044260	BP	6.62E-35	cellular macromolecule metabolic process
Cardiovascular	GO:0055010	BP	1.33E-06	ventricular cardiac muscle tissue morphogenesis
Dermatological	GO:0008544	BP	1.77E-10	epidermis development
Developmental	GO:0031017	BP	0.0017	exocrine pancreas development
Grey	GO:0032502	BP	2.09E-06	developmental process
Hematological	GO:0050817	BP	4.92E-35	coagulation
Immunological	GO:0002376	BP	6.54E-29	immune system process
Metabolic	GO:0006629	BP	3.91E-10	lipid metabolic process
multiple	GO:0006281	BP	2.93E-08	DNA repair
Muscular	GO:0061061	BP	0.151	muscle structure development
Neurological	GO:0007626	BP	5.00E-04	locomotory behavior
Nutritional	GO:0007631	BP	3.47E-05	feeding behavior
Ophthamological	GO:0007601	BP	3.70E-34	visual perception
Skeletal	GO:0035108	BP	0.0215	limb morphogenesis
