# Supplementary material for: NOA: a cytoscape plugin for network ontology analysis
Source: Bioinformatics. 2013 Jun 7;29(16):2066–7. doi: 10.1093/bioinformatics/btt334 (PMC3722524; doi:10.1093/bioinformatics/btt334)
Supplement: Supplementary Data [file supp_btt334_S5.pdf]

Supplementary Table S5

Node-based, All network, Corrected				
Disease	GO ID	Type	P-value	Description
Bone	GO:0010979	BP	0.0004	regulation of vitamin D 24-hydroxylase activity
Cancer	GO:0022402	BP	1.70E-13	cell cycle process
Cardiovascular	GO:0008016	BP	3.08E-09	regulation of heart contraction
Connective_tissue_disorder	GO:0030199	BP	0.0064	collagen fibril organization
Dermatological	GO:0008544	BP	1.09E-13	epidermis development
Developmental	GO:2000112	BP	0.0007	regulation of cellular macromolecule biosynthetic process
Ear_Nose_Throat	GO:0007605	BP	6.93E-26	sensory perception of sound
Endocrine	GO:0042445	BP	4.25E-09	hormone metabolic process
Gastrointestinal	GO:0008206	BP	6.07E-07	bile acid metabolic process
Grey	GO:0032502	BP	1.71E-09	developmental process
Hematological	GO:0007599	BP	7.05E-18	hemostasis
Immunological	GO:0002376	BP	1.35E-40	immune system process
Metabolic	GO:0044281	BP	2.01E-48	small molecule metabolic process
multiple	GO:0007031	BP	9.15E-05	peroxisome organization
Muscular	GO:0061061	BP	3.93E-10	muscle structure development
Neurological	GO:0016265	BP	4.60E-12	death
Nutritional	GO:0007631	BP	1.93E-06	feeding behavior
Ophthamological	GO:0007601	BP	1.67E-71	visual perception
Psychiatric	GO:0046219	BP	0.0201	indolalkylamine biosynthetic process
Renal	GO:0006811	MF	0.0005	ion transport
Respiratory	GO:0007585	BP	5.15E-06	respiratory gaseous exchange
Skeletal	GO:0001501	BP	2.70E-05	skeletal system development
