# Supplementary figures and images for: NOA: a cytoscape plugin for network ontology analysis
Source: Bioinformatics. 2013 Jun 7;29(16):2066–7. doi: 10.1093/bioinformatics/btt334 (PMC3722524; doi:10.1093/bioinformatics/btt334)

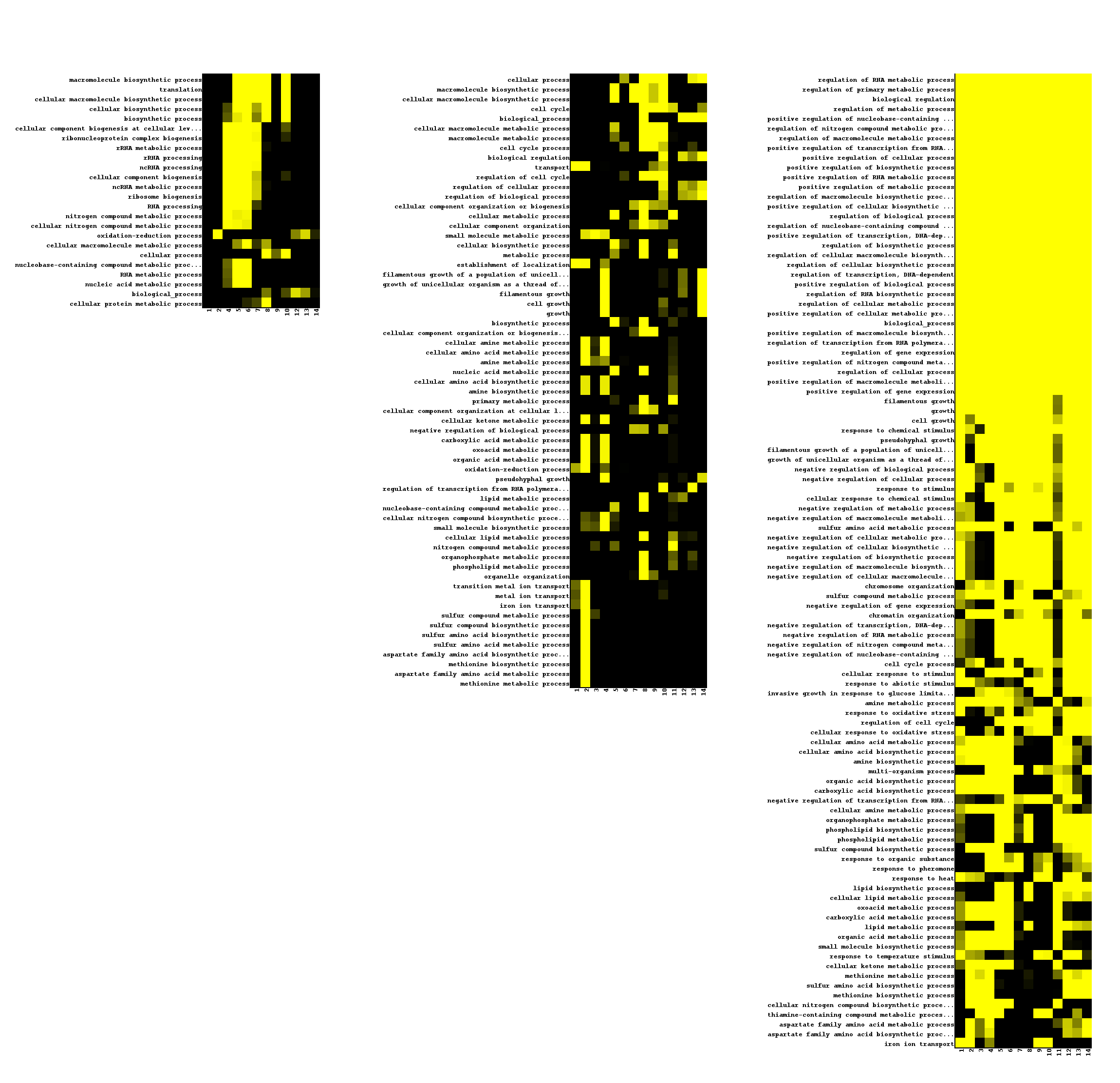

Supplement: Supplementary Data [file supp_btt334_S7.png]
